# Tibia Adamantinoma Resection and Reconstruction with a Custom-Made Total Tibia Endoprosthesis: A Case Report with 8-Year Follow-Up

**DOI:** 10.1155/2018/3656913

**Published:** 2018-06-06

**Authors:** Gilber Kask, Toni-Karri Pakarinen, Jyrki Parkkinen, Hannu Kuokkanen, Jyrki Nieminen, Minna K. Laitinen

**Affiliations:** ^1^Department of Orthopaedics and Traumatology, Unit of Musculoskeletal Surgery, Tampere University Hospital, Teiskontie 35, 33521 Tampere, Finland; ^2^Fimlab Laboratories, Arvo Ylpön katu 4, 33520 Tampere, Finland; ^3^Division of Plastic Surgery, Helsinki University Central Hospital, Topeliuksenkatu 5, 00260 Helsinki, Finland; ^4^Coxa Hospital for Joint Replacement, Biokatu 6, 33520 Tampere, Finland

## Abstract

This case study describes a total tibia resection and reconstruction with a custom-made endoprosthetic replacement (EPR) and a long-term, 8-year follow-up. The patient underwent a total tibia adamantinoma resection in 2009. Reconstruction was performed with a custom-made total tibia EPR, where both the knee joint and ankle joint were reconstructed. Two muscle flaps, latissimus dorsi free flap and a pedicled medial gastrocnemius flap, were used for soft tissue reconstruction. The patient returned to normal life as a kindergarten teacher, without complications for eight years. This case demonstrated the importance of successful multidisciplinary teamwork in close collaboration with industry. In our best knowledge, no over 2 years of follow-up of total tibia replacement reports have been published.

## 1. Introduction

Adamantinoma is a rare malignant bone tumor, accounting for approximately 0.1–0.5% of all primary bone tumors [1]. There is a slight male predominance [1–3]. Tibia is involved in 85–90% of cases, but the other sites, including the fibula, ulna, femur, humerus, and radius, have been also reported [2, 4].

There have been no definitive guidelines for a treatment of adamantinoma. The preferred treatment is surgical management with wide margins, reconstruction if necessary and possible, or amputation [1]. Methods of limb salvage with reconstruction include distraction osteogenesis, allografts, vascularized fibular autografts, nonvascularized autogenous bone grafts, or endoprosthetic reconstructions (EPRs) [5]. Chemotherapy and radiotherapy in adamantinoma treatment have not been shown to be effective [2, 6, 7], and therefore surgery remains the only curative option. After successful surgery with wide margins, the overall 10-year survival rates vary from 82% to 87% [1, 6]. In the literature, reported limb salvage rate for long bone adamantinoma patients is about 84% [3] and amputation compared with the limb preserving surgery has not been proved to improve survival rate [5].

Reconstruction after resection surgery for adamantinoma, in long bones, is dependent on the site and size of tumor. In the most common site, tibia, reconstruction is often EPR which is associated with a high rate of complications (48%) [3]. One of the most serious complication in EPR surgery is a periprosthetic joint infection (PJI). Deep infection in EPR around the knee is reported to be 4–45% [8]. Furthermore, limb reconstruction with extensive customized endoprosthesis is associated with an even higher incidence of serious complications. PJI is the main indication for a secondary amputation in EPR of the proximal tibia [9].

In cases where tumor affects the entire tibia, a wide excision with a clear margin is achievable with knee disarticulation, or higher amputation, or with a total tibia resection and reconstruction. In the current literature, only two cases of total tibia reconstruction have been published [10, 11]. One total EPR had a short follow-up of two years without early complications, and one case was reconstructed with a total tibia allograft, subsequently ending in a complication leading to amputation. To our best knowledge, no midterm or long-term results over 2 years of follow-up of total tibia replacement case reports have been published.

This study describes a case of complete tibia resection and total tibia custom-made EPR, with a long-term follow-up and functional outcome. This case study highlights the rarity of the reconstruction, multidisciplinary team work, and the good long-term functional result.

## 2. Case Presentation

**Figure 1:**
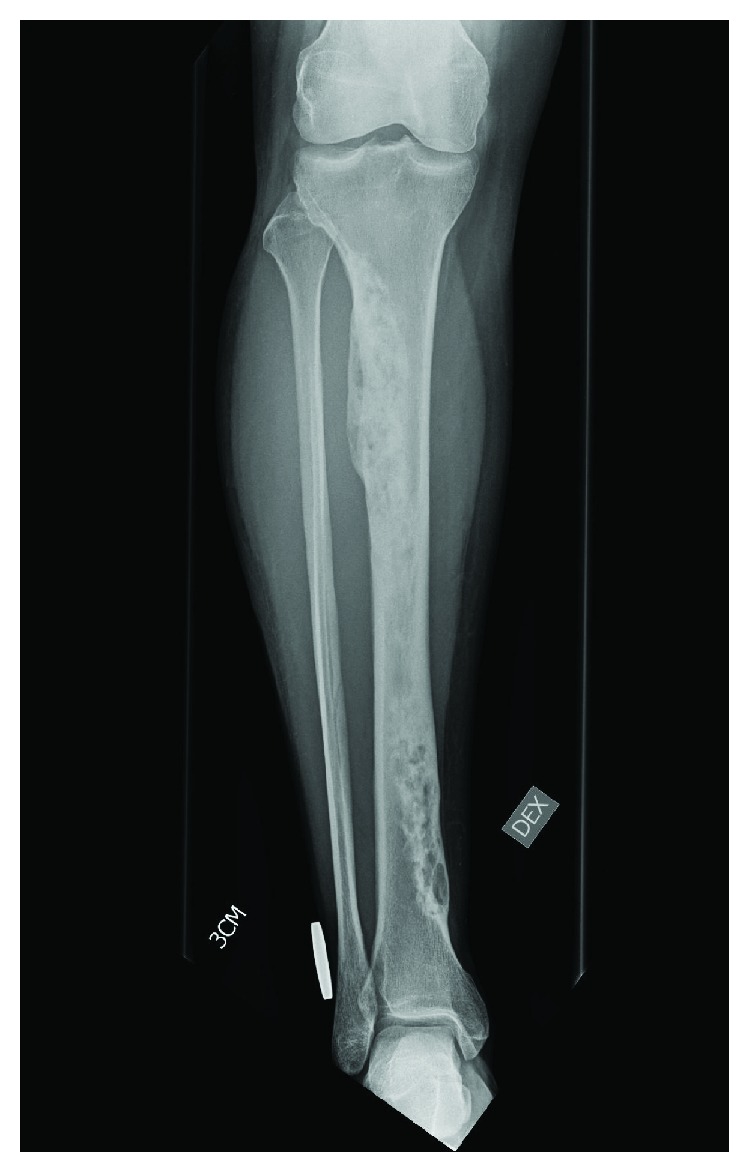
Preoperative X-ray from a right tibia. A plain X-ray from a right tibia with an intraosseal tumor that extended from 4 cm below the knee joint proximally to about 4 cm from the ankle joint distally.

A 48-year-old female with known breast carcinoma was screened for possible dissemination with whole-body computed tomography (CT) and a bone scintigraphy scan. The bone scan revealed a tumor in the entire right tibia. The patient reported no symptoms from the tibia tumor. A plain X-ray and magnetic resonance image (MRI) confirmed an intraosseal tumor that extended from 4 cm below the knee joint proximally to about 4 cm from the ankle joint distally (Figure 1). An open biopsy confirmed an adamantinoma histology. Different treatment options were thoroughly discussed with the patient, including a lower leg amputation with disarticulation of the knee, a total tibia resection and reconstruction with a tibia allograft, or a custom-made tibia EPR, which was eventually selected.

**Figure 2:**
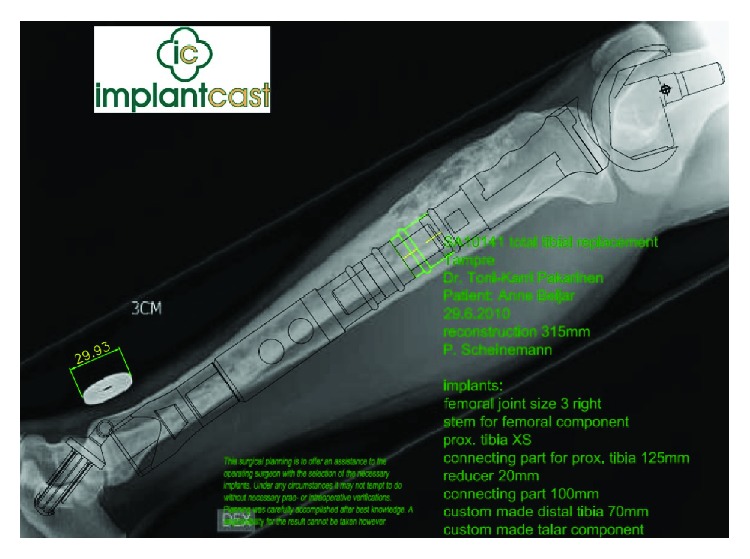
The preoperative planning of a custom-made total tibia endoprosthesis. The preoperative planning for total tibia resection and reconstruction with a custom-made, silver-coated, modular endoprosthesis of the Modular Universal Tumor and Revision System.

**Figure 3:**
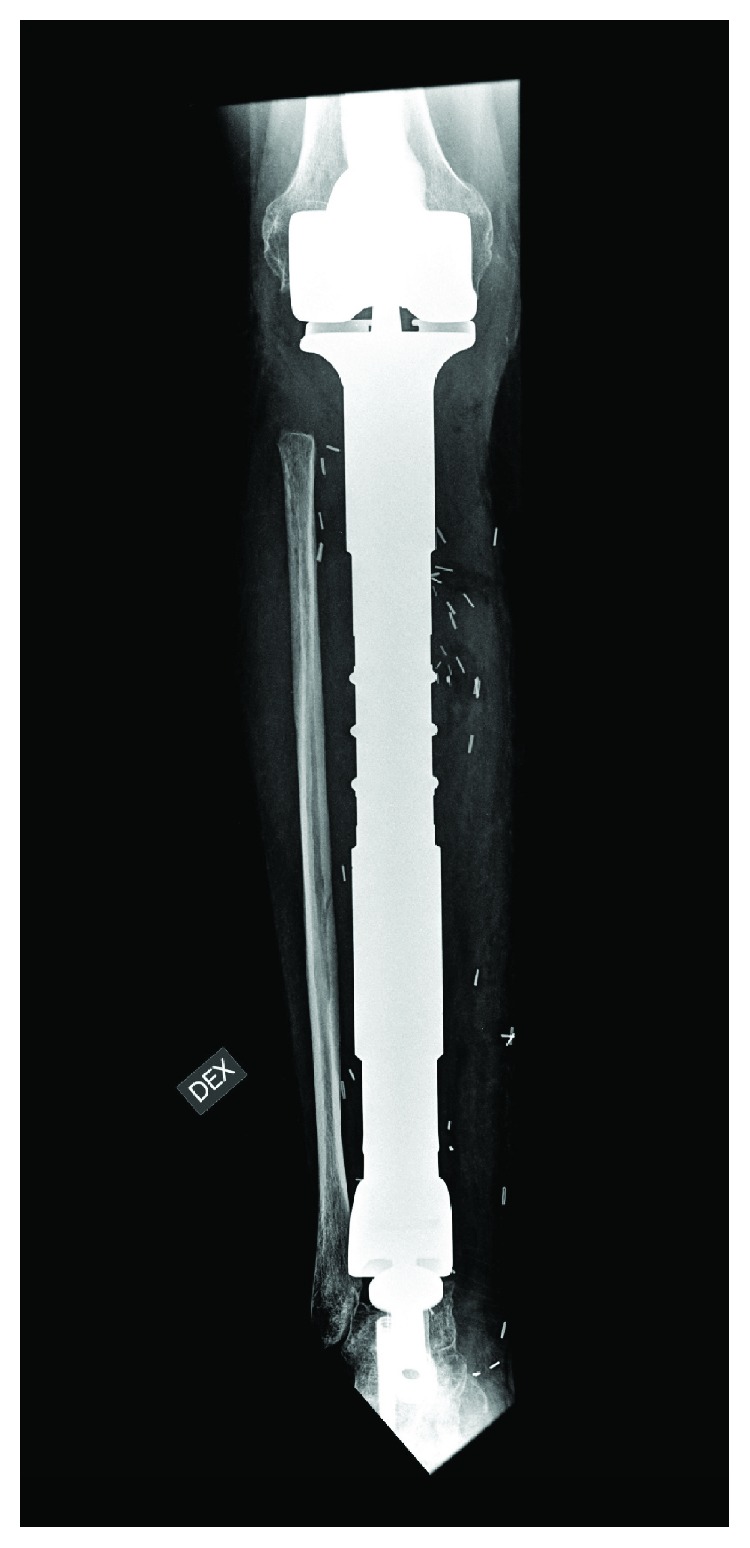
Postoperative X-ray from a right tibia. The knee joint was reconstructed with a metal-on-poly articulation with a (unique) metal-on-metal hinge mechanism, and the ankle joint was reconstructed with a metal-on-poly hinge joint with a talar replacement, stabilized with a trans-talar and trans-calcanear hydroxyapatite-coated stem. A supplementary screw was used to add stability in the subtalar joint.

**Figure 4:**
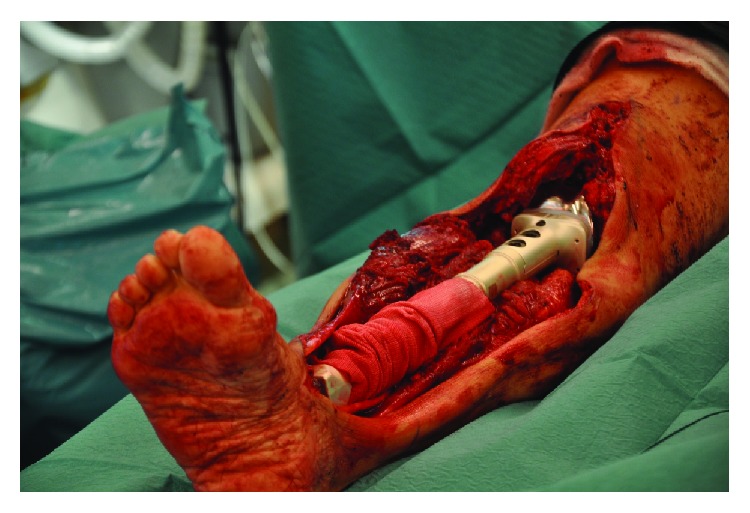
Perioperative image of the total tibia resection and reconstruction. The tibia tumor is totally resected, and the tibia is replaced with a custom-made endoprosthesis. The endoprosthesis was enveloped in a Trevira tube to facilitate the attachment of soft tissues and the patella tendon.

The tumor was resected with an extensive anteromedial approach, and the defect was reconstructed with a custom-made, silver-coated, modular endoprosthesis of the Modular Universal Tumor and Revision System (Implantcast®, Buxtehüde, Germany) (Figure 2). The knee joint was reconstructed with a metal-on-poly articulation with a (unique) metal-on-metal hinge mechanism (Figure 3). The ankle joint was reconstructed with a metal-on-poly hinge joint with a talar replacement, stabilized with a trans-talar and trans-calcanear hydroxyapatite-coated stem. A supplementary screw was used to add stability in the subtalar joint. The endoprosthesis was enveloped in a Trevira (Implantcast®) tube to facilitate the attachment of soft tissues and the patella tendon (Figure 4). A microvascular latissimus dorsi musculocutaneous flap was anastomosed to the tibia artery (end-to-side) and concomitant vein and wrapped around the prosthesis to avoid dead space and allow tension-free closure. In addition, a medial gastrocnemius muscle flap was transposed to cover the patellar tendon region; this was covered with a meshed split-thickness skin graft.

A histological analysis of the resected specimen showed an adamantinoma that had spread throughout the entire tibia and the margins were wide: an unaffected periosteum as an anatomic barrier and a minimum of clear soft tissue margin of 3 mm. Due to intracortical location of the tumor, no massive muscle excision was needed.

The knee joint was immobilized in extension for 6 weeks to facilitate patella ligament attachment to the tube. Then, the joint was gradually mobilized. After 6 months, the patient's gait was almost normal, with mild limping. Local MRI and chest radiographs performed during the 8 years of follow-up showed no signs of local recurrence or distant metastasis. No loosening of the stem or other mechanical problem was reported. In a routine follow-up, in addition to radiographs, we used a local MRI with a metal artifact reduction sequence (MARS) technique [12], which enables to observe local recurrences of the tumor around the massive titanium endoprosthesis.

Eight years postoperatively, the patient had most of the time no pain and could mobilize freely. The patient resumed working full-time as a kindergarten teacher, and she has maintained her previous active lifestyle (except downhill skiing). On her latest follow-up visit (at 8 years), the knee range of motion was 0–105 degrees, ankle dorsiflexion was 5 degrees, and ankle flexion was 35 degrees. In the latest follow-up visit, we used 4 patient-related outcome (PRO) measures. The Musculoskeletal Society Tumor Score (MSTS) was 77%; the Oxford Knee score (OKS) was 35/48; the Toronto Extremity Salvage Score (TESS) was 80/100; and the 15D was 0.87/1.

**Figure 5:**
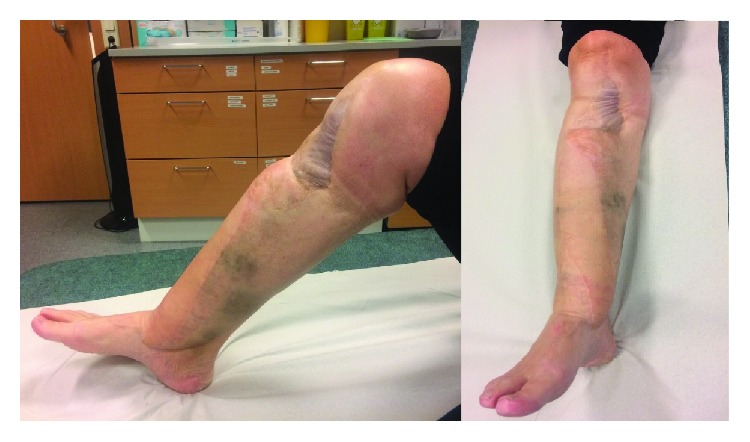
Patient's right lower extremity image in an 8-year follow-up visit. The silver coating created a mild, local skin argyria pigmentation, with cosmetic discomfort.

The metal-on-metal prosthesis caused an increase in metal ion concentrations: cobalt was 6 ppb and chromium was 8 ppb. The silver coating created a mild, local skin argyria pigmentation, with cosmetic discomfort [13] (Figure 5).

## 3. Discussion

Due to the subcutaneous location of the tibia and its close proximity to vital neurovascular and musculotendinous structures, limb salvage surgery can be difficult to achieve in tibia bone malignancies. Complication rates are higher in the proximal and distal tibia than at other locations, and they are highest with total tibia reconstructions [8]. PJIs, particularly in tibia locations, comprise the main indication for a secondary amputation. The 10-year implant survival rates are reported to be 40–74%, following tumor resection and reconstruction with different types of EPRs. However, those studies mostly investigated implant survival for fixed- or rotating-hinge knee prostheses, with some total femoral prostheses, but none investigated a total tibia replacement [9].

In our opinion, it is essential to use a well-vascularized flap after implant reconstruction. Muscle flaps have demonstrated good adherence to implants, and thus they resist seroma formation and lower the risk of infection. Technically, a free flap is relatively easy to use in the primary operation, because vascular structures are well exposed.

The soft tissue reconstruction is very important to prevent infection complications. In present case, a free latissimus dorsi flap was wrapped around the prosthesis to avoid dead space. Rotational medial gastrocnemius was used to cover the defects. In preankle area, it is very important to avoid dead space, cover the defects and get good vascularity, to prevent infection.

The antimicrobial activity of silver has gained interest in the orthopaedic community, especially among orthopaedic surgeons using megaendoprostheses. The main advantage of using silver-coated EPR includes the reduction of the incidence of PJI with a low level of toxicity [14–16]. Several side effects in silver-coated implants have been reported, including argyria, as seen in our patient, kidney and liver damage, leukopenia, and toxicity in neural tissues. The systemic effects have been reported with blood concentrations exceeding 300 ppb. In large clinical series of silver-coated EPR, silver levels in blood samples did not exceed 56 ppb and were considered nontoxic [17, 18]. Local asymptomatic argyria has been described to occur even in 23% of patients with silver-coated EPR [19].

Eight years postoperatively, the patient resumed working full-time as a kindergarten teacher, and except downhill skiing, she has maintained her previous active lifestyle. On her latest follow-up visit, the motions of knee and ankle ranges were good. In addition, we used 4 PRO measures in the latest follow-up visit (MSTS, OKS, TESS, and 15D). Based on these measures, patient had some limitations in walking and participating in usual leisure activities. She had mild pain from the knee, limping problems, and impossibility to kneel. In addition, patient had moderate difficulties in putting shoes on, gardening, getting in and out of the bath, walking upstairs and downstairs, and getting out of a car. The 15D questionnaire showed that patient had mild sleeping problems and mild sadness. In our opinion, patient's overall functional outcome was good.

Local MRI with a MARS technique enables to observe local recurrences of the tumor around the massive titanium endoprosthesis. This technique reduces the artifacts caused by endoprosthesis, improves the quality of the images at the periprosthetic region, and leads to reliable diagnosis of endoprosthesis-related problems [12].

Due to the rarity of a total tibia EPR, we could identify only one previous case report with a relatively short follow-up. In the literature case report [11], a total tibia EPR was performed for a patient with Ewing sarcoma. They described a 2-year follow-up, with no early-stage complications. In addition, another study has presented a 17-year follow-up of tibia replacement [20]. However, in this osteosarcoma case report, it was not a total tibia replacement as no total ankle joint replacement was made, since part of the distal tibia was not removed. Anterior part of the ankle is a critical location for complications, because of the movement and subcutaneous position of the joint. Also, one reconstruction with a total tibia allograft has been published [10]. In this report, eight months after the surgery, the patient reappeared with rapidly increased pain and the allograft was fractured. About 1 year after the initial diagnosis, a knee disarticulation was performed and the patient was supplied with an exoprosthesis. In this case, authors have discussed that using a prosthesis system instead of the allograft might have saved the limb of their patient [10].

The present report demonstrated that a complex total tibia EPR is feasible with a functionally good outcome. It is important to use a well-vascularized flap after implant reconstruction. Technically, a free flap is relatively easy to use in the primary operation. Muscle flaps have demonstrated good adherence to implants: they resist seroma formation and decrease the risk of infection. However, we emphasize that long-term success required a multidisciplinary team working closely in collaboration with the endoprosthesis industry.
